# Acute effect of electroacupuncture at the Zusanli acupoints on decreasing insulin resistance as shown by lowering plasma free fatty acid levels in steroid-background male rats

**DOI:** 10.1186/1472-6882-9-26

**Published:** 2009-08-01

**Authors:** Rong-Tsung Lin, Chung-Yuh Tzeng, Yu-Chen Lee, Wai-Jane Ho, Juei-Tang Cheng, Jaung-Geng Lin, Shih-Liang Chang

**Affiliations:** 1 Department of Internal Medicine, Division of Endocrinology and Metabolism, Tungs' Taichung Metro Harbor Hospital, Taichung County, Taiwan, Republic of China; 2 Department of Orthopedics, Taichung Veterans General Hospital, Taichung City, Taiwan, Republic of China; 3 Department of Acupuncture, China Medical University Hospital, Taichung City, Taiwan, Republic of China; 4 Department of Medicinal Botanicals and Health Care, Da-Yeh University, Chunghwa County, Taiwan, Republic of China; 5 Department of Pharmacology, College of Medicine, National Cheng Kung University, Tainan City, Taiwan, Republic of China; 6 Graduate Institute of Chinese Medical Science, China Medical University, Taichung City, Taiwan, Republic of China; 7 School of Chinese Medical Science, China Medical University, Taichung City, Taiwan, Republic of China

## Abstract

**Background:**

Insulin sensitivity has been enhanced by electroacupuncture (EA) in rats, but the EA phenomenon in an insulin resistant state is still unclear. This study reports the use of a large dose of prednisolone to evaluate the effects of EA in a state of insulin resistance.

**Methods:**

The plasma levels of free fatty acids (FFAs) were estimated in steroid-background rats (SBRs) and compared with those in healthy rats treated with normal saline. In addition, plasma glucose and endogenous insulin levels were assayed to calculate the homeostasis model assessment (HOMA) index. Intravenous glucose tolerance test (IVGTT) was carried out to compare glucose tolerance. The SBRs were randomly divided into EA-treatment and non-EA treatment groups and 15-Hz EA was applied to the bilateral Zusanli acupoints to investigate its effects on insulin resistance. In addition to an insulin challenge test (ICT) and IVGTT, the plasma levels of FFAs were measured and western blot was performed to help determine the effects of EA on the insulin resistant state.

**Results:**

The plasma levels of FFAs increased markedly in SBRs, the HOMA index was markedly higher, and glucose tolerance was impaired. EA improved glucose tolerance and insulin sensitivity by decreasing the plasma levels of FFAs. Further, the insulin signaling proteins (IRS1) and glucose transporter isoform protein (GLUT4) in skeletal muscle inhibited by prednisolone recovered after EA.

**Conclusion:**

Insulin resistance was successfully induced by a large dose of prednisolone in male rats. This insulin resistance can be improved by 15 Hz EA at the bilateral Zusanli acupoints, as shown by decreased plasma levels of FFAs.

## Background

Insulin resistance is a metabolic disorder caused by decreased biological responses to insulin [1]. Insulin signaling is a cascade of events initiated when insulin binds to the alpha-subunits of its surface receptor. The receptor beta-subunits then autophosphorylate, leading to activate tyrosine phosphorylation of insulin receptor substrates (IRSs) [2]. The binding of the regulatory subunit of phosphatidylinositol 3-kinase (PI3K) to IRSs activates PI3K and Akt, which are also called protein kinase B (PKB). The activation of this process is necessary for insulin to regulate glucose transporter 4 (GLUT4) protein translocation from the cytosol to the membrane for glucose transportation in skeletal muscle [3]. Theoretically, insulin resistance may be due to one or more of the following: (a) deficiency or defects in insulin receptors, (b) inactivation of GLUT4 proteins, or (c) deficiency of GLUT4 proteins [4]. This study measures the IRS1 and GLUT4 levels of EA and non-EA samples for both steroid background and saline groups to evaluate the possible effects of EA on insulin resistance.

Most patients with type 2 diabetes mellitus (DM) exhibit tissue insensitivity to insulin [5]. This disorder is related to genetic factors, and may be aggravated by other factors including a sedentary lifestyle, abdominal visceral obesity and a high-carbohydrate diet [6]. Other studies show that many circulating insulin inhibitors, such as anti-insulin antibodies, free fatty acids (FFAs), growth hormones, and glucocorticoids, are involved in the reduction of insulin sensitivity [7,8].

Most glucocorticoids are produced in the adrenal cortex. They have multiple functions, but mainly increase hepatic gluconeogenesis and inhibit peripheral glucose uptake in both muscle and adipose tissues. In adipose tissue, excess glucocorticoids lead to lipolysis, releasing FFAs and inducing insulin resistance [9,10] by inactivating GLUT4 translocation from the cytosol to the cell membrane for glucose uptake [11].

Researchers have indicated that glucocorticoids can inhibit glucose uptake at one or more steps along the insulin signaling pathway at the cellular level. Dexamethasone impairs insulin signaling and glucose transport by depleting IRS1, PI3K, and protein kinase B in primary cultured rat adipocytes [12]. In a previous study on the relationship of insulin resistance with blood pressure, chronic low-dose subcutaneous dexamethasone (2 ug/day) was given to Wistar rats for 4 weeks to induce insulin resistance [13]. In addition, another study showed that glucocorticoids induce the redistribution of fat from the periphery to the central abdominal compartment and increase visceral lipolysis, elevating plasma triglyceride (TG) and FFA levels. Reducing muscle glucose uptake also lowers glucose transportation activity [14].

Although some drugs can improve insulin resistance [15], the goal of many researchers is the development of new, more effective alternative therapies with fewer side effects. Acupuncture treatment is a component of traditional Chinese medicine (TCM), and has been used for over two thousand years. The commonly used acupoints for DM-like syndromes include Pishu (BL20), Geshu (BL17), Zhongwan (CV12), Sanyinjiao (SP6), Neiguan (PC6), and Zusanli (ST36) [16]. Researchers have recently begun using electroacupuncture (EA) instead, as it combines traditional needle acupuncture with an electrical current passing through the needles into the acupoints to produce hypoglycemic responses. Research suggests that EA at different frequencies causes the release of endogenous opioid peptides that activate specific receptors [17]. Researchers have also examined opioid receptors in the pancreas to determine their roles in the regulation of plasma glucose concentrations [18,19]. In addition, beta-endorphins enhance insulin secretion [20]. Applying EA to the Zhongwan acupoint induces the secretion of beta-endorphins, leading to insulin-dependent lowering plasma glucose action in diabetic rats [21].

Acupuncture can also lower plasma FFAs and lipid levels by altering citrate or glucose metabolism in the liver [22]. Moreover, EA at the Zhongwan (CV12) acupoint of *Psammomys obesus *produces a prolonged and sustained hypoglycemic effect by elevating insulin sensitivity [23]. However, the mechanisms by which EA improves insulin resistance remain unclear. The aim of this study is to determine if a single EA treatment can inhibit the development of glucocorticoid-altered insulin sensitivity and exploring the mechanisms of EA by way of assaying plasma FFAs and insulin signal proteins.

The experiments in this study applied intravenous glucose tolerance test (IVGTT) and insulin challenge test (ICT) to evaluate the effect of EA on insulin resistant steroid-background rats (SBRs). In addition, whole-animal insulin resistance was measured using the homeostasis model assessment (HOMA) index [24]. Finally, this study evaluates insulin signal proteins to investigate the mechanisms by which EA improves the insulin resistance of SBRs.

## Methods

### Animal Models

Male Wistar rats weighing 300–340 g and aged 8–10 weeks were used in this study. Steroid-induced insulin resistant rats were produced by intraperitoneal injection (i.p.) of 40 mg/kg prednisolone after the rats had fasted for 12 hours. Insulin resistance after the prednisolone injection was confirmed by intravenous glucose tolerance test and the HOMA index. These steroid background rats were prepared for evaluating the effect of EA on insulin resistance. The rats were maintained in the Animal Center of China Medical University, Taiwan, under a 12:12 h light-dark cycle (light on at 6:00 a.m.) in a temperature controlled room (25 ± 1°C). Food (Purina Rat Chow) and water were available ad libitum. All animals were anesthetized with 40 mg/kg pentobarbital (MTC Company, Canada) i.p. before all the experiments in this study. Animals were treated in accordance with the National Institute of Health (NIH) Guide for the Care and Use of Laboratory Animals, and this protocol was approved by an ethical committee in China Medical University, Taichung, Taiwan.

### Evaluating methods of insulin resistance in SBRs

At first, fasting normal male rats (n = 22) were randomly divided into a steroid group (SG) and a control (normal saline) group (CG). The SG rats were treated with a single intraperitoneal injection of large-dose prednisolone (40 mg/kg) dissolved in normal saline. The CG rats were treated with an intraperitoneal injection of the same volume of normal saline. Blood samples were drawn from the femoral veins at 30, 60, and 90 min post-treatment to estimate the plasma FFA levels of both groups. The plasma FFA levels (meq/l) were determined using a non-esterified fatty acid kit (Randox Laboratories, Canada Ltd.) and automatic spectrophotometers (COBAS system). Whole-animal insulin resistance was assessed using the HOMA index, as described previously [24]. This procedure involves the simultaneous evaluation of the fasting plasma insulin levels (μU/ml) multiplied by the fasting plasma glucose (mmol/l) and divided by 22.5. Endogenous plasma insulin levels (pmol/l) were determined using a commercial kit (Roche Elecsys systems) and the electrochemiluminescence immunoassay (ECLIA) method. Plasma glucose levels were measured using a spectrophotometer system (COBAS). At the end of the test, plasma insulin and glucose levels were assayed to calculate differences in the HOMA index between the two rat groups.

To confirm insulin resistance by IVGTT, fasting normal male rats (n = 26) were randomly divided into SG and CG groups. After injection of prednisolone i.p., IVGTT was processed by intravenous injection of 1 mg/kg glucose into the femoral veins of the SG group rats under anesthesia. The same IVGTT process was carried out on the CG rats. During the IVGTT, blood samples were drawn from the femoral veins of the rats at 0, 15, 30, 60, and 90 min after injections for plasma glucose assay in each group. The changes in the plasma glucose levels were then compared between the SG and CG groups.

### Electroacupuncture

In the EA experiment, the bilateral Zusanli acupoints located at the anterior tibia muscle near the knees were identified based on previous studies [25,26]. After adjusting the EA apparatus to 15 Hz/10 mA (Han's Healthronics Likon, Taipei, Taiwan), acupuncture needles (0.5 inch/32 gauge) were inserted 2–5 mm into the muscle layer of the selected acupoints. The positively charged (red pole) clip was connected to the right needle and the negatively charged (black pole) clip was connected to the left needle.

### Evaluating the effects of EA on SBRs

Another experiment was conducted to evaluate the effects of EA on insulin resistant SBRs and the changes in plasma insulin levels. Fasting normal male rats (n = 16) were first randomly divided into EA and non-EA groups. The two groups rats were then treated with prednisolone as described above, and allowed to rest for 90 min. The insulin resistant SBRs then received EA for 60 min under anesthesia, and were then compared with the non-EA group. An IVGTT was performed to compare the changes in plasma glucose level between the EA and non-EA groups at 0, 15, 30, 60, and 90 min post-treatment. Endogenous plasma insulin levels (pmol/l) were also estimated in the two group rats at 0, 15, 30, 60, and 90 min post-treatment. Further study of ICT was conducted to evaluate the effects of EA on plasma glucose levels and FFAs levels during an insulin challenge test on insulin resistant SBRs. The two groups rats (n = 18) were treated with prednisolone as described above and allowed to rest for 90 min. The SBRs were then randomly divided into EA and non-EA groups. The EA group rats received EA for 60 min under anesthesia, and then compared with the non-EA group. During ICT, 1 IU/kg i.p. of regular insulin (NovoNordisk Company, Denmark) was applied to both rat groups to compare plasma glucose and FFAs levels at 0, 30, and 60 min post-treatment.

In western blot analysis, fasting normal male rats (n = 18) were randomly divided into EA, non-EA (receiving the same treatments as described above), and normal saline (did not receive EA and prednisolone) groups. Blood samples were taken at the beginning and end of EA to determine the levels of plasma glucose and insulin for calculating the HOMA index. These blood samples were also assayed of plasma levels of FFAs, the percentage change (%) was calculated between the beginning and end of EA. At the end of EA, a part of gastrocnemius muscles in the back of the lower legs of the rats were cut off as sample for analysis of insulin signal proteins (IRS1, GLUT4), and actin served as a control. The muscle samples were homogenized in buffer solution before centrifugation at 13000 rpm. The obtained supernatant was used to estimate the amount of protein using an assay kit (Bio-Rad Laboratories, CA., USA). This supernatant (protein) was added to 4× loading dye, and boiled for 15 min at 95°C for denaturing. This process produces separating (8%) and stacking gel. Then, protein (90 ug/ml) in the buffer solution was loaded into each well for electrophoresis. Proteins were electrophoretically transferred to polyvinylidene difluoride membranes at 4°C. The membranes were then blocked with 5% nonfat dry milk in phosphate buffered saline (PBS) for 1 hr at room temperature and incubated with the specific primary antibodies (Santa Cruz Biotechnology, Inc, CA., USA). After the membranes were washed in a buffer containing 0.1% Tween 20 in 1× PBS, blots were incubated with a horseradish peroxidase-linked specific second antibody (Santa Cruz Biotechnology, Inc) followed by enhanced chemiluminescence detection using ECL reagent plus (PerkinElmer Life Sciences, Inc., USA). Band intensities were quantified by densitometry to observe the target proteins.

### Statistical Analysis

Results were calculated as a percentage change of the initial value according to the formula (Xi-Xt) × 100/Xi, where Xi is the initial level and Xt is the level after treatment. Statistical analysis was performed using SPSS analytical software. Data was expressed as mean ± SEM, and repeated measures analysis of variance (ANOVA) was performed to analyze plasma glucose and other variables. The source of significant differences was determined by post hoc comparison. The non-parametric Mann-Whitney test or Student's *t*-test was also applied to compare the differences between the two independent groups. A *p*-value less than 0.05 was considered statistically significant.

## Results

### Elevating insulin resistance in rats after prednisolone administration

After treatment with a large dose of prednisolone, the rats' plasma levels of FFAs gradually increased from 30 to 90 min. The SG group exhibited significantly higher plasma levels of FFAs at 90 min than the CG group. The percentage increase in plasma levels of FFAs at 60 min for the SG group was 38%, which was higher than the 11% increase in the CG group. At 90 min, the percentage increase in the SG group was 66%, while it was only 15% (*p *< 0.05) in the CG group (Table 1).

**Table 1:** Plasma FFA levels in normal Wistar rats treated with normal saline or prednisolone

Time (n = 11)	30 min	60 min	90 min
CG: Normal Saline	1.23 ± 0.5	1.29 ± 0.4	1.25 ± 0.5
SG: Prednisolone	1.01 ± 0.2, a	1.36 ± 0.2, b	1.64 ± 0.3, c *

Values given as mean ± SEM (meq/l) after treatment; n = number of animals; CG = control group; SG = steroid group; letters a, b, and c indicate statistical significance compared with each other using ANOVA with LSD post-hoc test: a < b < c, *p *< 0.05. Data from each group were compared using the non-parametric Mann-Whitney test. *p < 0.05

**Figure 1:**
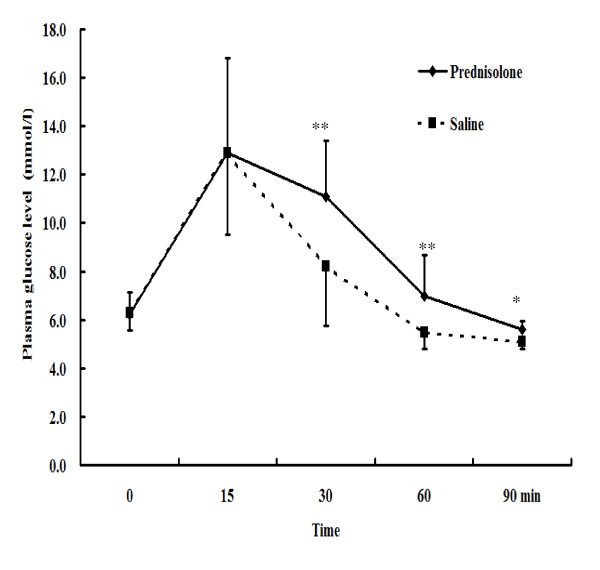
**Intravenous glucose tolerance tests (IVGTTs) to evaluate the insulin resistance in SBRs rats**. The plasma glucose levels of SBRs and the control group were compared using an independent *t*-test. *p < 0.05; **p < 0.01.

In addition, the SG exhibited a higher HOMA index than the CG group at 90 min (Table 2). Although the plasma levels of glucose were not significantly different between the two groups, the plasma levels of insulin in the SG group increased markedly at 90 min after the injection of prednisolone (Table 2). During the IVGTT, the SG had higher plasma glucose levels than the CG group from 30 to 90 min (Figure 1). The plasma glucose level at 30 min was 8.2 ± 2.4 mmol/l in the CG group, which was markedly lower than the 11.1 ± 2.3 mmol/l result in the SG group (*p *< 0.01). At 60 min, the plasma glucose level was 5.5 ± 0.7 mmol/l in the CG group, which was still lower than the 7.0 ± 1.7 mmol/l result in the SG group (*p *< 0.005). This trend remained unchanged at 90 min (*p *< 0.05).

**Table 2:** Homeostasis model assessment (HOMA) index of Wistar rats treated with normal saline or prednisolone 40 mg/kg i.p.

After 90 min (n = 8)	Glucose (mmol/l)	Insulin (pmol/l)	HOMA index
CG: Normal Saline	5.7 ± 0.6	0.27 ± 0.2	1.7 ± 1.2
SG: Prednisolone	5.8 ± 0.8	0.84 ± 0.4 *	7.29 ± 3.0 *

Values are given as mean ± SEM after treatment, n = number of animals. Data from each group were compared using the non-parametric Mann-Whitney test. *p < 0.05

### Effect of EA on SBRs evaluated by IVGTT

**Figure 2:**
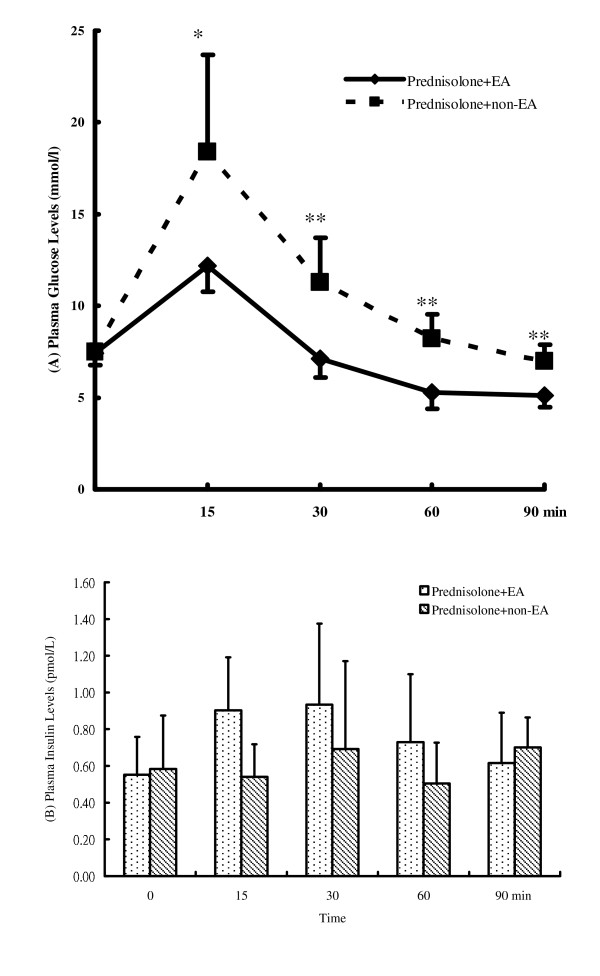
**(A) Plasma glucose levels and (B) plasma insulin levels for steroid background rats undergoing IVGTT**. Prednisolone + EA group = SBRs receiving EA for 60 min at the bilateral Zusanli acupoints. Prednisolone + non-EA group = SBRs without EA. *p < 0.05; **p < 0.01, EA vs. non-EA at the same time point.

The IVGTTs in this study revealed significant changes in plasma glucose lowering effect in the EA group compared with the non-EA group from 15 to 90 min post-treatment (Figure 2(A)). Figure 2(A) shows that the increase in plasma glucose levels after the injection of glucose was lower in the EA group than in the non-EA group. The plasma level of glucose at 90 min was 5.1 ± 0.6 mmol/l in the EA group, which was markedly lower than the 7.0 ± 0.9 mmol/l result in the non-EA group (*p *< 0.005). The differences in plasma levels of insulin between the EA and non-EA groups were not statistically significant at 90 min (Figure 2B).

### Effects of EA on SBRs evaluated by ICT

Table 3 shows that the ICT revealed a higher percent decrease in glucose in the EA group than that in the non-EA group. At the end of the test, the percentage decrease in glucose in the EA group was 77%, while that in the non-EA group was only 42% (*p *< 0.01). At the same time, the EA group had lower plasma levels of FFAs than the non-EA group.

**Table 3:** Effects of EA on plasma glucose and free fatty acids levels during an insulin challenge test in steroid background rats (SBRs)

Glucose mmol/l (n = 9)	0 min	30 min	60 min
	
Non-EA	6.3 ± 0.7	4.7 ± 1.2	3.8 ± 1.3
EA	6.3 ± 0.6	2.2 ± 0.7 **	1.4 ± 0.4 **
FFA meq/l (n = 9)			
Non-EA	1.1 ± 0.2	1.4 ± 0.3	1.4 ± 0.2
EA	1.2 ± 0.3	0.7 ± 0.3 **	0.7 ± 0.2 **

Values are given as mean ± SEM after 12 hours of fasting and 30 min after giving prednisolone; n = number of animals, EA = experimental group which received electroacupuncture, non-EA = control group without electroacupuncture. **p < 0.01 vs. control group at the same time.

### Effects of EA on SBRs evaluated by HOMA index and plasma FFA levels

The EA group in this study exhibited a lower HOMA index and a lower percentage of elevation in plasma FFAs compared with the non-EA group at 60 min after initiating the test in SBRs (Table 4). Although the plasma levels of glucose did not change significantly after a single dose of prednisolone in the non-EA group compared with the group treated with normal saline, the EA group exhibited a significantly lower plasma level of glucose. No differences in plasma insulin levels appeared between the EA and non-EA groups, but both groups showed higher plasma insulin levels than the group treated with normal saline alone (Table 4).

**Table 4:** Effects of EA on the homeostasis model assessment index in Wistar rats 60 minutes after treatment with a single large dose of prednisolone

Group (n = 6)	Glucose (mmol/l)	Insulin (pmol/l)	FFA (0–60 min) %	HOMA index
Normal Saline	6.5 ± 0.7, A_1_	0.27 ± 0.2, B_2_	17 ± 31, B_3_	1.8 ± 1.6, B_4_
EA (SBRs)	4.6 ± 0.6, B_1_	0.65 ± 0.2, A_2_	16 ± 20, B_3_	3.3 ± 1.1, B_4_
Non-EA (SBRs)	6.3 ± 0.3, A_1_	0.66 ± 0.2, A_2_	72 ± 31, A_3_	5.1 ± 1.0, A_4_

Values are given as mean ± SEM after treatment; n = number of animals, FFA = free fatty acids, HOMA = homeostasis model assessment, EA = electroacupuncture, SBRs = steroid background rats. Percentage (%) change from 0 to 60 min is shown in the column labeled FFA (0–60 min) %. The data for these four variables were compared using one way ANOVA. A_X _> B_X_, the least significant difference post hoc was taken; *p *< 0.05, the variable of "X = 1 to 4" represents the different indexes.

### Effect of EA on the insulin signaling protein expression of SBRs

**Figure 3:**
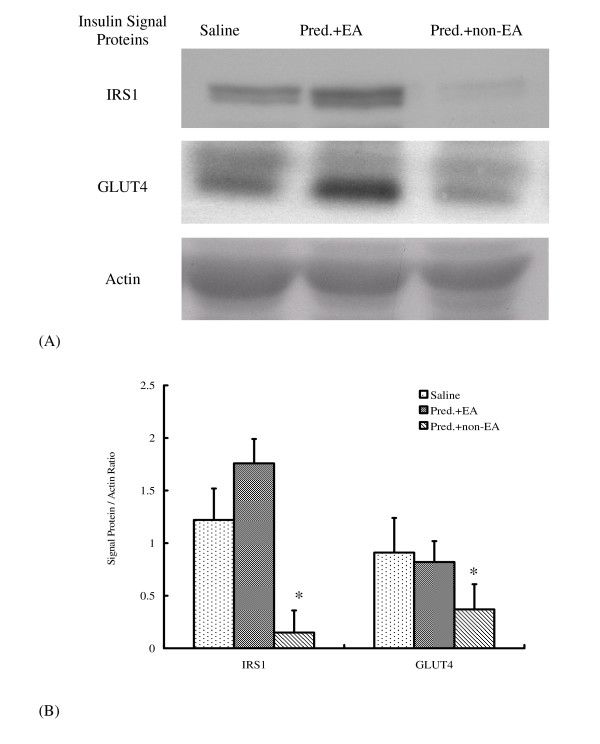
**(A) Insulin signaling proteins assayed using western blot. (B) All signal protein values were calculated as a ratio of signal protein to actin**. The ratios were compared within the groups: (1) Saline group: control treated with normal saline only (2) Pred. + non-EA: SBRs not treated with EA (3) Pred. + EA: SBRs treated with EA for 60 min. The one way ANOVA was used to compare the means among groups *p < 0.05 was considered statistically significant vs. saline group.

Significantly lower IRS1 and GLUT4 protein expression occurred in rats in the non-EA group receiving prednisolone 60 min post-treatment. No significant differences in IRS1 or GLUT4 appeared between the group treated with normal saline only and the group of SBRs that received EA treatments (Figure 3). The inhibition of IRS1 and GLUT4 proteins in SBRs was not seen in rats treated by EA.

## Discussion

According to traditional Chinese medicine theory, different acupoints have different effects [27]. Previous studies showed that EA stimulation at the bilateral Zusanli acupoints produces a greater hypoglycemic response than stimulation at the Zhongwan acupoint in normal rats [25,28]. Also, using 15 Hz EA produces a better hypoglycemic effect than 2 Hz EA [29]. Therefore, this study used 15 Hz EA on the bilateral Zusanli acupoints to explore its effects on enhancing insulin sensitivity. The purpose of this study is to evaluate whether or not EA stimulation can improve insulin sensitivity by decreasing plasma FFAs levels in steroid background rats with insulin resistance.

Glucocorticoids are important in the etiology of insulin resistance through down-regulation of insulin signal proteins. In a dose-dependent manner, steroids cause desensitize the glucose transport system in muscle [30,31]. In addition, glucocorticoid-related hyperglycemia is also dose dependent [32]. Although there is still insufficient studies to support the animal model of insulin resistance caused by a large dose of prednisolone, this study presents a faster method and a new animal model of elevating short-term insulin resistance using a large dose of prednisolone as background in normal Wistar rats, and using IVGTT, HOMA index and FFA for the realization of steroid induced insulin resistance. Further, this study shows the effects of EA in SBRs with an insulin resistant state.

The data produced by comparing experiments between SG and CG in this study shows that a large dose of prednisolone effectively produces insulin resistance. We cannot rule out the possibility that higher insulin levels (Table 2) are the result of impaired insulin clearance rather than increased compensatory secretion. However, the expression of insulin signal proteins (IRS1 and GLUT4) was inhibited in SBRs, which is why prednisolone produces insulin resistance.

This study determines the hypoglycemic effects of EA in SBRs using IVGTTs and ICTs to investigate ways to decrease insulin resistance or inhibit the development of insulin resistance. During the IVGTT experiment, the EA group exhibited lower plasma glucose levels than the non-EA group in SBRs (Figure 2A). The EA group and the non-EA group did not exhibit significantly different plasma insulin levels in these SBRs (Figure 2B). This may be because insulin secretion was stimulated by exogenous glucose or because prednisolone was applied to the SBRs during the IVGTT. Thus, EA had no further effect on increasing insulin secretion. This result is compatible with previous studies, which showed that 15 Hz EA on the bilateral Zusanli acupoints significantly lowers plasma glucose levels during an IVGTT in normal Wistar rats. In addition, the EA group and the non-EA group showed no differences in plasma insulin levels in normal Wistar rats [33]. In summary, 15 Hz EA on the bilateral Zusanli acupoints can enhance plasma glucose lowering effects in both normal Wistar rats and SBRs, not through an increase in insulin secretion, but by enhancing the insulin sensitivity of the target organ. Also, a non-insulin dependent hypoglycemic mechanism may be considered in 15 Hz EA on the bilateral Zusanli acupoints in our unpublished results. The results in Table 4 illustrate this inference. The EA group and the non-EA group had the same plasma insulin levels, but the EA group had lower plasma glucose levels than the non-EA group. Although Figure 1 and 2 report these results using the parametric statistical method with higher deviation, this study also uses nonparametric statistical methods, such as the Mann-Whitney test, to double check these results, obtaining the same trend as the results using by parametric statistical method in this study.

In the ICT, hypoglycemic effects were increased in the EA group compared with the non-EA group of SBRs. This test helps reveal whether or not EA enhances insulin sensitivity in SBRs given exogenous insulin. The results of this study agree with previous studies, which showed that 15 Hz EA on the bilateral Zusanli acupoints induces significantly higher hypoglycemic activity in normal Wistar rats and streptozotocin (STZ)-induced diabetic rats than in experimental animals without EA during an ICT [33]. In addition, more hypoglycemic effects were observed at 60 min in SBRs which received EA during ICT (from 6.3 ± 0.6 to 1.4 ± 0.4 mmol/l below the normal range) than those without EA (from 6.3 ± 0.7 to 3.8 ± 1.3 mmol/l; Table 3). Furthermore, there was a marked decrease in FFA levels (from 1.2 ± 0.3 to 0.7 ± 0.2 meq/l below the normal range) at 60 min in the EA group of SBRs during ICT than the SBRs without EA (Table 3). In summary, a decrease in plasma FFA levels may play an important role in improving insulin sensitivity when using 15 Hz EA on the bilateral Zusanli acupoints during ICT. Furthermore, EA reversed the low expression of insulin signal proteins (IRS1, GLUT4) in SBRs (Figure 3) and reduced the HOMA index in SBRs to a level similar to that in the group that received normal saline (Table 4). Therefore, it is reasonable to suppose that EA can improve insulin resistance in SBRs by decreasing the plasma FFA levels and recovering insulin signal proteins. This implies that there is an increase in insulin activity after using EA, although further study of the insulin signal change mechanisms is required.

## Conclusion

Using EA at the bilateral Zusanli acupoints, this study tests the hypothesis that EA can improve insulin resistance by lowering plasma free fatty acids in SBRs male rats. Further clinical trials in humans should be undertaken to obtain more evidence for this hypothesis. However, a large dose of prednisolone increased insulin resistance, and this study successfully establishes a method of evaluating the effect of EA in improving insulin sensitivity in an insulin resistant state. In addition, results show that 15 Hz EA on the bilateral Zusanli acupoints may improve insulin resistance by decreasing the plasma FFA levels and recovering the expression of insulin signal proteins (IRS1 and GLUT4), which enhances insulin activity. Thus, EA should be considered a method of alternative therapy for prednisolone induced insulin resistance.

## Competing interests

The authors declare that they have no competing interests.

## Authors' contributions

**SLC **and **RTL **carried out the study design, experimental work, data collection and interpretation, literature review, and manuscript preparation. **CYT **and **YCL **provided assistance with applying for the supporting grant. **WJH **provided an excellent research environment and participated in discussion and coordination. **JTC **and **JGL **supervised the work, evaluated the data, and corrected the manuscript for publication. All authors read and proved the final manuscript.

## Pre-publication history

The pre-publication history for this paper can be accessed here:


